# Application of d-SPE before SPE and HPLC-FLD to Analyze Bisphenols in Human Breast Milk Samples

**DOI:** 10.3390/molecules26164930

**Published:** 2021-08-14

**Authors:** Tomasz Tuzimski, Szymon Szubartowski

**Affiliations:** 1 Department of Physical Chemistry, Medical University of Lublin, Chodźki 4a, 20-093 Lublin, Poland; 2 Doctoral School of Medical University of Lublin, Medical University of Lublin, Chodźki 7, 20-093 Lublin, Poland

**Keywords:** bisphenols, human breast milk samples, high-performance liquid chromatography (HPLC), fluorescence detector (FLD), dispersive solid phase extraction (d-SPE)/solid phase extraction (SPE), BADGE (bisphenol A diglycidyl ether) and its derivatives

## Abstract

In this study, we propose a simple, cost-effective, and sensitive high-performance liquid chromatography with fluorescence detection (HPLC-FLD) for the simultaneous determination of seven bisphenols (bisphenol F (BPF), bisphenol E (BPE), bisphenol B (BPB), BADGE (bisphenol A diglycidyl ether), BADGE∙2H_2_O, BADGE∙H_2_O, BADGE∙2HCl) in human breast milk samples. The dispersive solid phase extraction (d-SPE) coupled with solid phase extraction (SPE) procedure performed well for the majority of the analytes with recoveries in the range 57–88% and relative standard deviations (RSD%) of less than 9.4%. During the d-SPE stage, no significant matrix effect was observed thanks to the application of different pairs of salts such as zirconium-dioxide-based sorbents (Z-Sep or Z-Sep +) and primary secondary amine (PSA) or QuEChERS Enhanced Matrix Removal-Lipid (EMR-Lipid) and PSA. The method limits of quantification (mLOQs) for all investigated analytes were set at satisfactory low values in the range 171.89–235.11 ng mL^−1^. Analyte concentrations were determined as the average value from human breast milk matrix samples. The results show that the d-SPE/SPE procedure, especially with the application of EMR-Lipid and PSA, could be used for further bisphenol analyses in human breast milk samples.

## 1. Introduction

Bisphenol A (BPA) is one of the most commonly produced xenoestrogens worldwide. It is mainly used for the production of epoxy resins, polycarbonates, and thermal paper and is present in commonly used products such as toys, water pipes, and food packaging materials such as plastic bottles or cans [1]. Due to its toxicity, the European Food Safety Authority (EFSA) analyzed scientific studies and set the maximum specific migration limit (SML) for BPA at 0.05 mg per kilogram of food (mg/kg) in 2018, updating the previous level set in 2011 [2]. Moreover, the European Union (EU) has prohibited the use of BPA in baby bottles [3]. This regulation has forced producers to introduce BPA analogues like bisphenol S (BPS), bisphenol F (BPF), bisphenol E (BPE), and bisphenol B (BPB) to the market. These analogues can be found in environmental and biological samples and exhibit similar or even higher levels of toxicity [4,5,6,7,8,9,10]. For this reason, the EU has limited the SML for one of the BPA analogues (BPS) to 0.05 mg per kilogram of food [11].

Bisphenol A diglycidyl ether (BADGE) is a product of the reaction of BPA with epichlorohydrin [12,13,14]. It can be found in canned foods, beverages, paints, and adhesives [12]. BADGE can form derivatives under certain storage conditions. Hydrolyzed compounds such as BADGE∙H_2_O and BADGE∙2H_2_O are formed during contact with aqueous and acidic foodstuffs [12,13,14]. Chlorinated derivates like BADGE∙HCl, BADGE∙2HCl, and BADGE∙HCl∙H_2_O are formed in the presence of hydrochloric acid, salty foodstuffs and during thermal coating treatment [13].

BADGE and its derivates show estrogenic and androgenic activity. In addition, in vitro studies have indicated that they have genotoxic, cytotoxic, and reduced fertility effects [13,14]. Due to the proven toxicity of BADGE and its hydrolyzed and chlorinated derivates, Commission Regulation (EC) No. 1895/2005 has set specific migration limits (SMLs). The SML for the sum of BADGE, BADGE∙H_2_O, and BADGE∙2H_2_O must not exceed 9 mg/kg in food and food simulants, and the sum of BADGE∙HCl, BADGE∙2HCl, and BADGE∙HCl∙H_2_O in food and food simulants is limited to 1 mg/kg [15].

However, the current regulations are not sufficient and require constant updating. Therefore, it seems necessary to work on new methods of extraction and determination of bisphenols in various samples to control human exposure to these xenobiotics. The analysis of bisphenols remains a current challenge, an example of which is the ongoing program “Clarity-BPA” in the USA [16].

Solid-phase extraction (SPE) is a popular sample preparation method. It is commonly used for the determination of bisphenols in biological samples [17,18,19,20,21]. It is a relatively cheap and easy technique with high levels of efficiency and reliability. However, for some complex matrices like human breast milk, SPE can be insufficient. For this reason, combining SPE with other sample purification techniques is an interesting alternative for the determination of bisphenols in biological samples.

QuEChERS (quick, easy, cheap, effective, rugged, and safe) is a new sample preparation method that was developed in 2003 to monitor pesticide residue levels in food [22]. Due to its flexibility, QuEChERS has been used successfully for many types of analytes in various types of samples such as bisphenols in human breast milk [23,24,25,26,27]. Dualde et al. used the QuECHERS method and liquid chromatography-mass spectrometry (LC-MS) to quantify three bisphenols (BPA, BPS, and BPF) in 10 human breast milk samples. Concentrations ranged from 0.13 to 1.62 ng mL^−1^ [26].

Today, less expensive alternatives to mass spectrometry (MS) are still used for HPLC analysis. These alternatives are the diode-array detector (DAD) and the fluorescence detector (FLD), which can be used to determine bisphenol levels in food or biological samples [28,29,30,31,32,33,34,35].

Bisphenols are compounds consisting of two phenyl rings linked together. The log *P* of analyzed compounds in our study ranged from 2.1 to 4.6, as tabularized in Table 1. This means that bisphenols should dissolve better in organic, lipophilic solvents. All bisphenols shown in Table 1 comply with Lipinski’s rule of five (less than five proton donors, less than ten proton acceptors, molecular mass less than 500 Dalton, and log *P* below five). This means that the estimated levels of absorption and permeation through biological membranes might be very high [36], making bisphenols especially dangerous due to their toxicity.

Despite having similar chemical structures, compounds analyzed in our study exhibited different lipophilic properties. Therefore, it is particularly difficult to obtain high levels of recovery for each of them. For that reason, reliable extraction techniques and analytical methods should be developed for the identification and quantification of bisphenol residues in human breast milk samples, especially when dealing with nanogram per milliliter levels of analytes.

The analysis of bisphenol residue in human breast milk samples is still a challenging issue with respect to analytes due to the partially fatty nature (4–8% lipids) of the sample matrix. On one hand, some lipids are co-extracted, which might cause significant difficulties during subsequent analysis. On the other hand, some fat-soluble nonpolar analytes or more lipophilic compounds (such as BADGE and BADGE∙2HCl) might persist in the fatty part of the sample, leading to poor extraction efficiency.

The aim of this study was to develop a method that provides optimal recovery values for seven selected bisphenols while maintaining adequate purification of samples and a low matrix effect. In this study, we attempted to optimize the procedure to determine the concentrations of analytes other than those studied in our previous papers [23,24] such as BADGE and its derivates and BPE in human breast milk samples by HPLC-FLD.

In this study, we applied a novel sample treatment method that connects QuEChERS/d-SPE and SPE to purify human breast milk samples. Yang et al. [17] carried out sample treatment with SPE using Oasis PRiME HLB cartridges and achieved very high recovery levels. Despite the application of several variants of sample preparation prior to SPE (e.g., removing fat by *n*-hexane and freezing out fat), the matrix effect remained at a high level. Furthermore, after repeating this procedure [17], we obtained much lower recovery values than those declared by other authors. In previous studies, the QuEChERS/d-SPE method was used to prepare human breast milk samples with HPLC-DAD and HPLC-DAD-FLD [23,24]. The procedure was confirmed using LC-MS, and four analytes, BPS, BPB, BPA, and BPB, were detected and quantified in 27 human breast milk samples. The BPB concentration ranged from 10.6 to 581.1 ng in 5 mL human breast milk samples. Other analytes (BPS, BPA and BPF) were detected, but their concentrations were below the limit of quantification (LOQ) [24].

To the best of our knowledge, this method is the first to combine the advantages of d-SPE and SPE as extraction techniques with high-performance liquid chromatography coupled with a fluorescence detector (HPLC-FLD). This could aid in the identification and quantification of bisphenol residues in human breast milk samples.

## 2. Results and Discussion

### 2.1. Chromatographic and Detection Conditions (HPLC-FLD)

Bisphenol standards (see Table 1) were chromatographed under conditions based on a previously published method that was applied, after appropriate modifications, for the determination of selected bisphenols [24]. Separation of the seven bisphenols under investigation was performed in the Scherzo SM-C18 multimodal stationary phase using a simple mobile phase consisting of water and acetonitrile, both acidified with formic acid (50 mM HCOOH in water (component A) and 50 mM HCOOH in acetonitrile (component B)) in a gradient system, as described in the “Experiment” section. The applied gradient elution program allowed for the appropriate separation of the analytes under investigation in a single chromatographic run (less than 15 min). The developed chromatographic system was characterized by a satisfactory analytical performance. Identification of the analytes was accomplished based on their retention times. Retention times are presented in Table 2.

Thanks to signal amplification in the range 0–18, the FLD detector enables the analysis of analytes at very low concentration levels (e.g., picograms per milliliter). Additionally, thanks to its sensitivity and selectivity, only compounds with fluorescence properties can be detected on the FLD detector, which decreases the influence of interference, and the matrix effect is much lower than with the application of the DAD detector.

Performing an analysis with the FLD detector makes it possible to obtain four different chromatograms simultaneously at four optimal excitation and emission wavelengths for analytes. Therefore, it allows the most optimal conditions for the quantitative analysis of bisphenols to be chosen. During the experiments, as most of the interference of matrices was eluted in the first 6 min, we applied reinforcement from 6 to 15 min.

This optimized chromatographic system and detection technique allowed for the separation of the determined bisphenols from the remaining sample components. The detection conditions allowed for better detection of the analytes, which were separated from other constituents of the sample, and evaluation of the matrix effect in the sample.

### 2.2. Optimization and Validation of the HPLC-FLD Method

During the development of the method, we conducted experiments to optimize different stages. The chromatographic method proposed for the separation and quantification of the analytes was validated in terms of LOQs. Standard calibration curves for the analytes were constructed by plotting the analyte concentrations against the peak area (the details are described in Section 3.4.2. Linearity).

The calibration curves of the bisphenols under investigation showed satisfactory levels of linearity and a correlation between the concentration and peak area for the studied range with a determination coefficient of *R*^2^ ≥ 0.997.

The limits of quantitation (LOQs) were determined through the analysis of samples with known analyte concentrations and by establishing the minimum level at which an analyte could be quantified with acceptable levels of accuracy and precision [37].

The LOQ values of the analytes were determined by constructing calibration curves using methanol (Table 2).

Therefore, in order to determine the method LOQ (mLOQ) values of the analytes, calibration curves were constructed in the averaged matrix. In order to prepare an averaged milk matrix, samples were taken from nine lactating women and mixed to ensure homogeneity (Table 3).

Calibration curves were prepared for all concentrations of bisphenols in the averaged matrix of human breast milk samples. LODs, LOQs, and mLOQ values were calculated for all analytes as described by the formulae presented in the Experiment section.

To evaluate linearity, calibration curves with seven concentration points were prepared for each bisphenol. The calibration curves were constructed by analyzing average human breast milk matrix samples containing different concentrations of target bisphenols previously treated with the optimized d-SPE/SPE procedure. The retention times, equations of calibration curves (which were constructed for an average human breast milk matrix sample) obtained by means of the least-squares method for bisphenol standards, determination coefficients (*R*^2^), limits of detection (LODs), and limits of quantification (LOQs) are given in Table 2. For all bisphenols in the investigated range of concentrations, linear dependencies were obtained. The lowest LOD and LOQ values in the average human breast milk matrix sample were obtained for BPF and BPE.

### 2.3. Sample Preparations and Optimization of the d-SPE/SPE-Based Procedure

**Figure 1:**
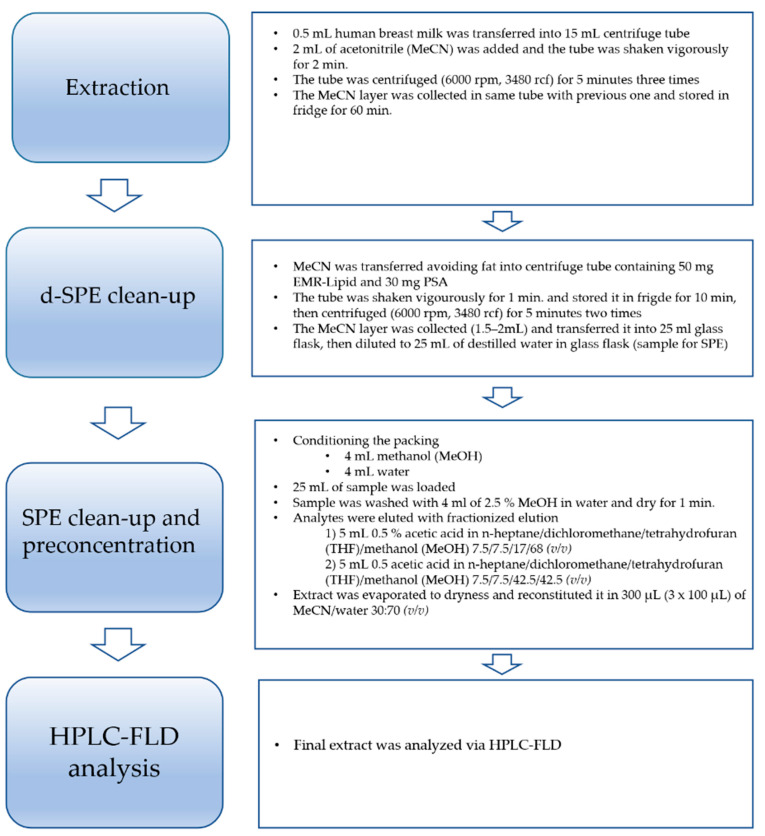
Flowchart of the developed d-SPE/SPE-based extraction procedure cleanup used for the detection of bisphenol residues in human breast milk samples.

The four-step sample preparation procedure consists of extraction and preconcentration, a d-SPE step, an SPE clean-up step, and HPLC-FLD analysis. A flowchart of the final procedure is presented in Figure 1.

The recovery, repeatability, and degree of chemical interference in the presence of the matrix were studied for the developed sample preparation procedure. The described d-SPE/SPE-HPLC-FLD procedure was applied for the analysis of human breast milk samples. For further quantitative and qualitative determination, incubation with ß-glucuronidase should be conducted at the beginning of the procedure.

#### 2.3.1. d-SPE/SPE-Based Sample Preparation Development

Bisphenols were extracted from human breast milk samples by liquid–liquid extraction, which is the first step of the described procedure. Acetonitrile, when applied as the extraction solvent (please see “Extraction” stage on the flowchart), facilitates the preparation of the milk proteins from a sample. This can be explained as a decrease in the dielectric constant of the milk sample, which increases the strength of the electrostatic interactions between the proteins. Additionally, the organic solvent displaces water from the hydrophobic regions of proteins, which results in more frequent aggregation of the proteins and subsequent precipitation from the sample [38]. Therefore, applying salting-out assisted liquid–liquid extraction enables substantial simplification of the matrix at the beginning of the described procedure by removing the vast majority of proteins and peptides that are present in milk samples. After this extraction step, a preconcentration stage was incorporated into the procedure to achieve lower mLOQs (Table 3). Method LOQs (mLOQs) were set at the minimum spiking level (ng mL^−1^) that could be quantified with acceptable accuracy and precision (i.e., for food samples: recovery in the range of 70–120% with an RSD of ≤15%). In our research, we analyzed bisphenols in biological samples. Biological samples (human breast milk samples) of limited amounts were collected from women. The details of the procedure used for the determination of mLOQs are clearly described in the Experiment section.

#### 2.3.2. Optimization of Dispersive Solid-Phase Extraction (d-SPE) before the SPE Procedure

Next, the cleanup step of the dispersive solid-phase extraction (d-SPE) method was optimized. For this purpose, four different sorbents were tested including relatively new commercially available sorbents such as zirconium-dioxide-based Z-Sep and Z-Sep +, EMR-Lipid (for the novel enhanced matrix removal of lipids), and primary secondary amine (PSA). Primary secondary amine (PSA) is a weak anion exchanger sorbent with the ability to remove sugars, organic acids, fatty acids, and polar pigments, while its chemical structure has a highly chelating effect. Z-Sep, Z-Sep +, and EMR-Lipid remove fatty and hydrophobic matrix interferences [38,39,40,41,42,43]. Zirconium-based dispersive phases have the ability to extract more fatty non-polar interferences (e.g., lipids) and pigments than traditional PSA and octadecyl (C18) sorbents and, therefore, in many cases, are associated with greater analyte recovery and better reproducibility.

The ratio of PSA to Z-Sep/Z-Sep +/EMR-Lipid was chosen during the optimization procedure (on the basis of previous experiments). The following approaches (with pairs of sorbents) concerning the extract cleanup step were evaluated: 50 mg Z-Sep and 30 mg PSA; 50 mg Z-Sep+ and 30 mg PSA; 50 mg EMR-Lipid, and 30 mg PSA. Extracts were also tested without d-SPE cleanup.

Section 2.4 describes the usefulness of the method for purifying matrices from interferences with simultaneous estimation of the recovery values of the analytes.

### 2.4. Recovery and Repeatability Studies

The percent average recovery and repeatability expressed as the RSD percent (*n* = 6) were studied in human breast milk samples for the final procedure (d-SPE cleanup using 50 mg EMR-Lipid and 30 mg PSA) at two spiking levels: 100 ng/mL (50 ng per sample) and at 250 ng/mL (125 ng per sample) (Table 4).

Different SPE procedures were tested in terms of their ability to isolate, extract, and concentrate the investigated bisphenols from human breast milk samples. Extraction was performed on Oasis HLB, 400 mg SPE columns. Before the traditional SPE step, dispersive solid-phase extraction was applied with different pairs of sorbents. The average recovery values obtained with the above combinations for human breast milk samples were partially satisfactory.

Both cleanup approaches, 50 mg Z-Sep or Z-Sep + and 30 mg PSA, exhibited similar recoveries, while the approach with 50 mg EMR-Lipid and 30 mg PSA provided higher recovery values. Thus, the results show that fatty and lipophilic interferences from human breast milk samples can be removed efficiently, especially with Z-Sep and Z-Sep + and with EMR-Lipid. Moreover, the effectiveness of the cleanup was also confirmed by taking the presence of interfering peaks on the chromatogram into consideration. The human breast milk extracts had lower backgrounds and provided optimal chromatograms with less interference after the d-SPE cleanup step (especially with 50 mg EMR-Lipid and 30 mg PSA). Taking these results into account as well as the simplicity of the routine analysis operation, the d-SPE cleanup step using 50 mg EMR-Lipid and 30 mg PSA was chosen to analyze the human breast milk samples. Thus, human breast milk samples were prepared according to the previously described and optimized d-SPE/SPE procedure.

#### Recovery Values Obtained after Optimization of the d-SPE/SPE-HPLC-FLD Procedure

The more hydrophobic analytes had the lowest recoveries, as exemplified by the series of BADGE and its derivatives. The lower recoveries for the more hydrophobic analytes were probably caused by more combinations of hydrophobic analytes within the fat phase of the sample, causing losses during the dispersive solid phase extraction step.

The lower recoveries for the more lipophilic analytes such as BADGE and BADGE∙2HCl may have been due to the use of sorbents (Z-Sep. Z-Sep +. EMR-Lipid) to remove the hydrophobic components of the sample along with the more hydrophobic analytes associated therewith.

Due to the low recovery values for the most lipophilic analytes, some modifications were made to the final procedure. As mentioned in the Section 3.6*. Optimization of the d-SPE/SPE-Based Extraction Procedure*, lipophilic solvents like 7.5% *n*-heptane and 7.5% dichloromethane were added to provide better extraction efficiency, especially for most lipophilic bisphenols (final version of procedure shown in Figure 1). The recovery values of the analytes were in the range 57 to 88% with RSD% values of less than 9.4% (see Table 4).

Finally, an important factor influencing the analysis of the bisphenols by the HPLC-FLD was the matrix effect, which results in an increase or suppression of the analytical signal. Usually, dilution of the sample reduces the matrix effect and the amount of matrix injected, achieving better peak shapes. In the present study, despite the significant concentrations of the final extracts (after drying and reconstitution in a small volume of eluent), satisfactory results were obtained. The results revealed satisfactory recoveries and clean chromatograms.

Due to the varied physicochemical properties of the analyzed bisphenols, resulting from their diverse structures, they had various affinities to the stationary phase of Scherzo SM C18, hence their varied elutions and the need to use a gradient with a pure organic modifier.

**Figure 2:**
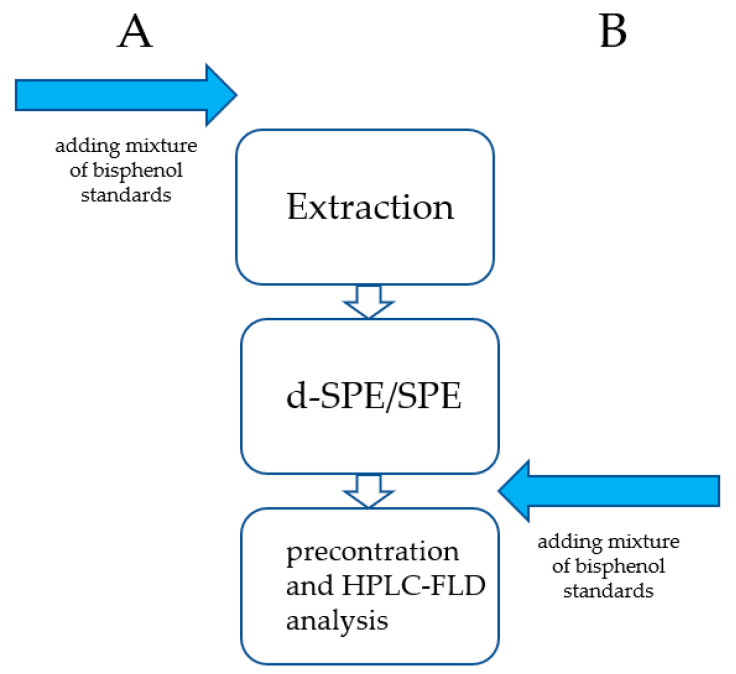
Graphical scheme of the calculation of the recovery values.

The described procedure including the recovery study (see Figure 2) is not yet optimal for all analytes, especially for BADGE.

Therefore, experiments in this area are continuing with the use of internal standards such as 4-phenylphenol (certified reference material), and we hope that the related studies will be published in the near future as a continuation of the experiments described in this paper.

### 2.5. Application of the Optimized Procedure to the Identification of Bisphenols in Human Breast Milk Samples by HPLC-FLD

The quantitative determination of selected bisphenols in human breast milk samples was performed under the same chromatographic conditions (please see the Experiment section). The identities of the analyte peaks in biological samples were confirmed by comparing their retention times with those of the relevant bisphenol standards.

**Figure 3:**
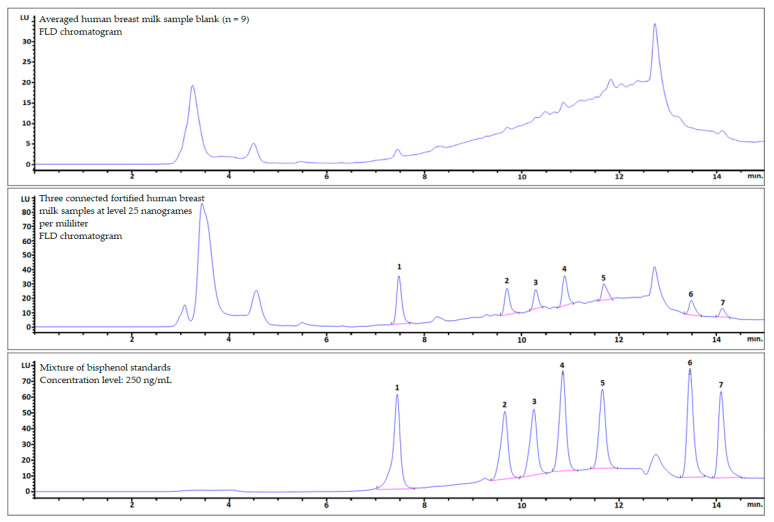
Comparison of FLD chromatograms of a human breast milk sample: blank (**top**); three connected fortified samples at 25 ng per milliliter (**middle**); and mixture of bisphenol standards (**bottom**).

The procedure optimized by us was used to analyze three human breast milk samples spiked at a level close to the mLOQ (25 ng per milliliter of sample). The samples were combined and concentrated into one sample for analysis. As shown in Figure 3 (see also Table 4), the sensitivity of the FLD allowed for the correct identification of all analyzed bisphenols and quantitative analysis of some analytes without MS. The obtained results seem to be reliable due to the low matrix effect (Figure 3, top).

## 3. Experiment

### 3.1. Chemicals and Reagents

The following standards used for the bisphenols under investigation were obtained from Sigma-Aldrich (Bellefonte, PA, USA): 3-[4-[2-[4-(2.3-Dihydroxypropoxy)phenyl]propan-2-yl]phenoxy]propane-1.2-diol (BADGE·2H_2_O, No. 1), bisphenol F (BPF, No. 2), bisphenol E (BPE. No. 3), 3-[4-[2-[4-(Oxiran-2-ylmethoxy)phenyl]propan-2-yl]phenoxy]propane-1.2-diol (BADGE·H_2_O, No. 4), bisphenol B (BPB. No. 5), 1-Chloro-3-[4-[2-[4-(3-chloro-2-hydroxypropoxy)-phenyl]propan-2-yl]phenoxy]propan-2-ol (BADGE·2HCl, No. 6), and 2-[[4-[2-[4-(Oxiran-2-ylmethoxy)phenyl]propan-2yl]phenoxy]methyl]oxirane (BADGE, No. 7). The standard purity indicated by the manufacturers for all of the reference standards of bisphenols was ≥98.0%

### 3.2. Solvents and Mobile-Phase Solutions

LC-MS grade methanol (MeOH), the gradient grade for liquid chromatography acetonitrile (MeCN) and formic acid were obtained from E. Merck (Darmstadt, Germany); LC-MS grade water was purchased from Sigma-Aldrich (St. Louis, MO, USA). Deionized water (0.07–0.09 mS cm^−1^) was produced in our laboratory using a Hydrolab System (Gdańsk, Poland). All analytical equipment including solvents and reagents was checked for bisphenol contamination prior to analysis by HPLC-FLD. Individual stock standard solutions were prepared in methanol and stored in screw-capped glass tubes in a refrigerator (+2 to +4 °C in the dark). A bisphenol standard mixture containing all of the analytes was prepared by combining suitable aliquots of each individual standard stock solution and diluting them with methanol. This was stored under the same conditions as individual stock standard solutions for up to two weeks. This mixture was used for calibration preparation as well as for the fortification of the human breast milk samples.

### 3.3. Apparatus and HPLC-FLD Conditions

An Agilent Technologies 1200 HPLC system with a quaternary pump and an autosampler that could thermostat samples was used for the LC analysis. Analytes were separated using a Scherzo SM-C18 150 mm × 4.6 mm column with a 3-µm particle size (Agilent Technologies, Wilmington, DE, USA). The column was thermostated at 22 °C. The mobile phase consisted of 50 mM HCOOH in water (component A) and 50 mM HCOOH in acetonitrile (component B) in a gradient elution: 0–10 min from 40% eluent B to 100% B; 10–14.5 min isocratic 100% B. The mobile phase flow rate was 0.4 mL/min.

In order to elute interferences of the matrix, before the next step of human breast milk sample analysis, the isocratic elution with 100% B as the mobile phase was applied for 15 min with a flow rate of 1 mL/min, and the next isocratic elution had the initial conditions.

FLD detection was carried out simultaneously at four different excitation wavelengths (225, 230, 235, and 240 nm). The emission wavelength was set at 300 nm.

### 3.4. HPLC-FLD Analysis and Method Validation

A validation study was performed using spiked human breast milk samples and included evaluation of the selectivity, linearity, limits of detection (LODs), limits of quantification (LOQs), mLOQ, matrix effects, extraction recovery, process efficiency, and precision and accuracy.

#### 3.4.1. Selectivity

The selectivity was evaluated by analyzing the human breast milk samples from different sources to investigate potential interferences with the signals of analytes. The extent of interferences originating from endogenous human breast milk sample components at the specific retention time of each analyte was evaluated through a comparison of an average blank human breast milk matrix sample (collected from nine women and then mixed) with the spiked average blank human breast milk matrix sample. HPLC analyses of bisphenol standards were repeated three times. The identification of bisphenols was accomplished on the basis of the retention times of the analytes.

#### 3.4.2. Linearity

The linearity of the method was studied by spiking the average blank human breast milk matrix sample with suitable amounts of bisphenol standards. Samples were prepared according to d-SPE/SPE and determined by the HPLC-FLD method described in the Experiment section. Solutions of the bisphenol standards were added to the average blank human breast milk matrix sample. Calibration curves for the LOD and LOQ values were constructed by analyzing bisphenol standards in methanol at six concentrations, from 10 to 500 ng/mL, using six replicates. The calibration curves were obtained by means of the least-squares method.

The calibration curves for mLOQ values were constructed by analyzing the spiked average blank human breast milk matrix sample at nine concentrations, over the range 10–500 ng/mL, using six replicates. The calibration curves were obtained by means of the least-squares method.

The limits of detection (LODs) and limits of quantification (LOQs) obtained for bisphenols were calculated according to the formulas LOD = 3.3 (SD/S), and LOQ = 10 (SD/S), where SD is the standard deviation of the response (peak area) and S is the slope of the calibration curve. HPLC analyses of bisphenol standards were repeated three times.

The method LOQs (mLOQs) were set at the minimum spiking level (ng mL^−1^) that could be quantified with acceptable levels of accuracy and precision. The method limits of detection (mLODs) and quantification (mLOQs) obtained for bisphenols were calculated according to the formulas mLOD = 3.3 (SD/S) and mLOQ = 10 (SD/S), where SD is the standard deviation of the response (peak area) and S is the slope of the calibration curve.

The identification of bisphenols was accomplished on the basis of the retention times of the analytes.

#### 3.4.3. Calculation of Extraction Recovery

The mLOQs were set as the minimum spiking levels (ng/mL) that could be quantified with acceptable levels of accuracy and precision. Extraction recovery was evaluated at a concentration level of 150 ng/mL according to the following formula:

Recovery (%) = A/B × 100%

where A is the peak area of the determined analyte in the sample after the procedure (explanation regarding spiking sample: the proper concentration of bisphenol was obtained after adding a solution of the standard to the average blank human breast milk matrix sample before starting the procedure shown in Figure 1); and B is the peak area of the determined analyte in the post-extraction sample (explanation regarding the post-extraction sample: the proper concentration of bisphenol was obtained after adding a solution of the standard to the final extract of the average blank human breast milk matrix sample after the SPE step and before evaporation of the final extract).

The relative standard deviation values were calculated as follows:

$$\mathrm{RSD}\%=\frac{\mathrm{Standard}\;\mathrm{deviation}\;\mathrm{of}\;\mathrm{the}\;\mathrm{recovery}\;\left(\%\right)}{\mathrm{mean}\;\mathrm{recovery}\;\left(\%\right)}\times 100\%$$

### 3.5. Dispersive Solid Phase (d-SPE) Salts and Solid Phase Extraction (SPE) Sorbents

Single-packaged sorbents used to prepare the sets (their mixtures) used during the d-SPE stage such as clean primary secondary amine (PSA), Z-Sep and Z-Sep +, QuEChERS were obtained from Sigma-Aldrich (Bellefonte, PA, USA); Enhanced Matrix Removal–Lipid (EMR-Lipid) was obtained from Agilent (Folsom, CA, USA).

### 3.6. Optimization of the d-SPE/SPE-Based Extraction Procedure

Human breast milk samples (0.5 mL) were transferred to 15 mL Falcon centrifuge tubes and spiked with an appropriate amount of a mixture of bisphenol standards and 2 mL of acetonitrile (MeCN). Tubes were shaken vigorously for two minutes and centrifuged for 5 min, three times (6000 rpm, 3480 rcf). After centrifugation, the MeCN layer was transferred into the next tube and frozen for 1 h in the fridge (−23 °C).

For the d-SPE step, one of the three pairs of salts (50 mg Z-Sep and 30 mg PSA, 50 mg Z-Sep+ and 30 mg PSA, 50 mg EMR Lipid and 30 mg PSA) was weighed into a tube. For the final version of the procedure, the option with 50 mg EMR Lipid and 30 mg PSA was chosen. Then, MeCN was transferred into a tube with a salt pair to avoid frozen fat, shaken for 1 min, refrigerated for 10 min (−23 °C), and centrifuged for 5 min two times (6000 rpm, 3480 rcf).

The MeCN layer was transferred into a 25 mL glass flask and diluted to 25 mL of deionized water to prepare the sample for the SPE clean-up step. An Oasis HLB cartridge (400 mg sorbent per cartridge, 60 µm, Waters Corporation, Milford, MA, USA) was conditioned with 4 mL of methanol and 4 mL of water. Then, 25 mL of the sample was loaded. The cartridge was washed with 4 mL of 2.5% methanol in deionized water and dried for 1 min. Then, analytes were eluted with fractionized elution:

(1) 5 mL 0.5% acetic acid (CH_3_COOH) in *n*-heptane/dichloromethane/tetrahydrofuran (THF)/methanol (MeOH) 7.5/7.5/17/68 (*v*/*v*)
(2) 5 mL 0.5 acetic acid (CH_3_COOH) in *n*-heptane/dichloromethane/tetrahydrofuran (THF)/methanol (MeOH) 7.5/7.5/42.5/42.5 (*v*/*v*).

Two fractions were connected and then evaporated to dryness and reconstituted in 300 µL MeCN: water 30:70 (*v*/*v*) (3 × 100 µL).

### 3.7. Human Breast Milk Sample Collection

Human breast milk samples were obtained from patients from the Department of Obstetrics and Pathology of Pregnancy, Medical University of Lublin, Poland. Sample collection was conducted from October 2019 to March 2020. After washing the breasts with clean water, the mothers (or donors) collected the samples into glass containers using a BPA-free breast pump. All samples were collected in glass bottles and immediately analyzed or frozen at –23 °C until analysis. This study was approved by the ethics committee of the Medical University of Lublin, Poland (No. KE-0254/271/2018).

## 4. Conclusions

In this study, a dispersive solid-phase extraction before solid-phase extraction procedure (d-SPE/SPE) and high-performance liquid chromatography coupled with a fluorescence detector (HPLC-FLD) were combined to form a sensitive and rapid method for the determination of selected bisphenols in human breast milk samples.

The optimization strategy involved the selection of purification conditions by applying different pairs of sorbents for the cleanup step by dispersive solid-phase extraction (d-SPE) in order to achieve acceptable recoveries and low amounts of co-extractives in the final human breast milk extracts.

The identification of analytes was based on their retention times and the application of the fluorescence detection technique (FLD).

The method was evaluated in terms of its linearity, recovery, precision, and the method limit of quantification (mLOQ). The method presented excellent linearity in the tested concentration ranges with correlation coefficient values ≥0.9824 obtained from a calibration curve constructed through a least-squares linear regression analysis for all cases.

In comparison to previously published results [24], the proposed method has the following advantages:

(1) A 10-fold reduction in the sample volume (from 5 to 0.5 mL);
(2) Optimization of the d-SPE/SPE technique for the majority of the analyzed bisphenols;
(3) Optimal recovery values obtained for all analytes in the range 57 to 88% for seven bisphenols (compared to 41.5–115.9% in [24] for only four analytes) combined with a low matrix effect, ensuring the reliable identification and quantification of analytes;
(4) The sample volume of 0.5 mL enabled us to combine several milk samples from one woman, allowing for the identification and quantitation of the analytes in biological samples using a sensitive fluorescence detector (FLD); and
(5) Due to the use of HPLC-FLD, it was possible to identify and quantify bisphenols in human milk samples without having to confirm their identities using tandem mass spectrometry.

The method was validated, and the procedure was applied to samples spiked at 25 ng/mL.

Thus, the adaptation of a method for the simultaneous detection of bisphenols in human breast milk samples by combining the d-SPE/SPE procedure with coupling detection techniques (FLD) produces a simple, fast, and cost-effective analytical alternative.

The proposed d-SPE/SPE procedure is recommended for further analyses of bisphenols in small human breast milk samples.

## Figures and Tables

**Table 1:** Physicochemical properties of the selected bisphenols.

No.	Bisphenol	IUPAC Name	Chemical Structure	Molecular Weight ^1^ (g/mol)	Log *P* ^1^	Proton Donors ^1^	Proton Acceptors ^1^
1	**BADGE∙2H_2_O**	2,2-bis[4-(2,3-hydroxypropoxy)phenyl]propane	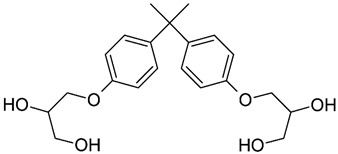	376.4	2.1	4	6
2	**BPF**	4-[(4-hydroxyphenyl)methyl]phenol	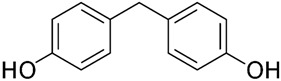	200.23	2.9	2	2
3	**BPE**	4-[1-(4-hydroxyphenyl)ethyl]phenol	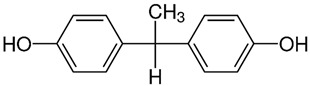	214.26	3.9	2	2
4	**BADGE∙H_2_O**	3-[4-[2-[4-(oxiran-2-ylmethoxy)phenyl]propan-2-yl]phenoxy]propane-1,2-diol	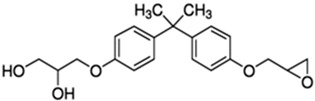	358.4	3.1	2	5
5	**BPB**	4-[2-(4-hydroxyphenyl)butan-2-yl]phenol	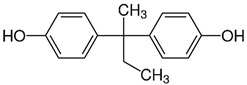	242.31	3.9	2	2
6	**BADGE∙2HCl**	1-chloro-3-[4-[2-[4-(3-chloro-2-hydroxypropoxy)phenyl]propan-2-yl]phenoxy]propan-2-ol	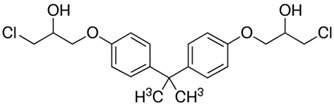	413.3	4.6	2	4
7	**BADGE**	2-[[4-[2-[4-(Oxiran-2-ylmethoxy)phenyl]propan-2-yl]phenoxy]methyl]oxirane	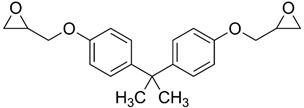	340.40	4.0	0	4

^1^ Data obtained from PubChem database.

**Table 2:** Validation parameters for the method: retention times, calibration curve equations (which were constructed using methanol), determination coefficients (*R*^2^), limits of detection (LODs), and limits of quantification (LOQs) obtained for the seven bisphenols by HPLC-FLD.

No.	Bisphenol	Retention Time, *t*r, Min	Concentration Range (ng mL^−1^)	λ (nm)	Linear Regression	Coefficient of Determination (*R*^2^)	LOD (ng mL ^−1^)	LOQ (ng mL ^−1^)
**1**	BADGE∙2H_2_O	~7.25	10–500	225	y = 0.9741x + 5.0885	*R*^2^ = 0.9988	8.59	26.03
				230	y = 1.4517x + 4.6832	*R*^2^ = 0.9989	8.05	24.38
				235	y = 1.7949x + 3.242	*R*^2^ = 0.9995	5.28	16.00
				240	y = 1.5781x + 9.9644	*R*^2^ = 0.9991	7.37	22.35
**2**	BPF	~9.30	10–500	225	y = 0.5699x − 1.6479	*R*^2^ = 0.9989	8.27	25.07
				230	y = 0.8048x − 1.357	*R*^2^ = 0.9993	6.44	19.50
				235	y = 0.992x + 4.1204	*R*^2^ = 0.9978	11.51	34.89
				240	y = 0.9516x + 2.286	*R*^2^ = 0.9998	3.77	11.42
**3**	BPE	~9.85	10–500	225	y = 0.3955x − 1.6879	*R*^2^ = 0.9994	7.15	21.66
				230	y = 0.5889x − 1.3777	*R*^2^ = 0.9991	8.31	25.20
				235	y = 0.7349x − 2.5296	*R*^2^ = 0.9998	3.56	10.79
				240	y = 0.7022x − 2.5784	*R*^2^ = 0.9998	4.42	13.39
**4**	BADGE∙H_2_O	~10.45	10–500	225	y = 1.1016x − 7.841	*R*^2^ = 0.9997	4.87	14.77
				230	y = 1.6107x − 10.732	*R*^2^ = 0.9998	4.37	13.23
				235	y = 1.894x − 9.433	*R*^2^ = 0.9996	5.48	16.60
				240	y = 1.7384x − 10.49	*R*^2^ = 0.9998	4.42	13.41
**5**	BPB	~11.20	10–500	225	y = 0.6156x + 3.7901	*R*^2^ = 0.9984	9.89	29.98
				230	y = 0.8606x + 3.2632	*R*^2^ = 0.9982	10.59	32.09
				235	y = 1.0132x + 5.9297	*R*^2^ = 0.9996	4.65	14.10
				240	y = 0.9414x + 4.3649	*R*^2^ = 0.9987	9.03	27.35
**6**	BADGE∙2HCl	~12.95	10–500	225	y = 0.7846x − 4.9256	*R*^2^ = 0.997	18.04	54.68
				230	y = 1.1371x − 5.2341	*R*^2^ = 0.9978	15.52	47.04
				235	y = 1.2953x + 1.0193	*R*^2^ = 0.9989	10.93	33.13
				240	y = 1.2299x + 0.9965	*R*^2^ = 0.9991	9.86	29.89
**7**	BADGE	~13.65	10–500	225	y = 0.6249x − 5.8007	*R*^2^ = 0.991	22.15	67.12
				230	y = 1.1797x − 24.386	*R*^2^ = 0.9824	20.33	61.62
				235	y = 1.4312x − 25.859	*R*^2^ = 0.9858	19.90	60.30
				240	y = 1.3158x − 20.146	*R*^2^ = 0.9892	15.45	46.83

**Table 3:** Method validation parameters used for fortified blank samples: retention times, calibration curve equations (constructed for the average human breast milk matrix sample), determination coefficients (*R*^2^), method limits of detection (mLODs), and method limits of quantification (mLOQs).

No.	Bisphenol	Retention Time (Min)	Concentration Range (ng mL^−1^)	λ (nm)	Linear Regression	Coefficient of Determination (*R*^2^)	mLOD (ng mL^−1^)	mLOQ (ng mL^−1^)
**1**	BADGE∙2H_2_O	~7.25	50–500	240	y = 0.901x + 21.716	*R*^2^ = 0.9905	56.72	171.89
**2**	BPF	~9.3	50–500	240	y = 0.518x − 9.5053	*R*^2^ = 0.9857	69.82	211.58
**3**	BPE	~9.85	50–500	240	y = 0.4142x − 9.8027	*R*^2^ = 0.9873	65.63	198.88
**4**	BADGE∙H_2_O	~10.45	50–500	240	y = 0.4695x − 3.6844	*R*^2^ = 0.9824	77.59	235.11
**5**	BPB	~11.20	50–500	240	y = 0.4459x − 12.885	*R*^2^ = 0.9824	77.55	234.99
**6**	BADGE∙2HCl	~12.95	50–500	240	y = 0.3002x + 6.5983	*R*^2^ = 0.9901	57.80	175.14
**7**	BADGE	~13.65	50–500	240	y = 0.2315x − 1.2545	*R*^2^ = 0.9889	61.37	185.96

**Table 4:** Mean recoveries (%) and relative standard deviations expressed as percentages (RSD%) for the mixture of bisphenols extracted by d-SPE/SPE using OASIS HLB (400 mg) cartridges.

**Recoveries Obtained for Fortification at 100 ng/mL Sample after the Procedure Shown in Figure 1.**																
**Bisphenol**	**Intra-Day Repeatability ^a^**						**Inter-Day** **Repeatability ^b^** **(*n* = 18)**		**Intra-Laboratory** **Reproducibility ^c^**						**Overall ^d^** **(*n* = 30)**	
**Name**	**Day 1 (*n* = 6)**		**Day 2 (*n* = 6)**		**Day 3 (*n* = 6)**				**Analyst 1 (*n* = 6)**		**Analyst 2 (*n* = 6)**		**Mean (*n* = 12)**			
	**Recovery %**	**RSD%**	**Recovery %**	**RSD%**	**Recovery (%)**	**RSD%**	**Recovery** **%**	**RSD%**	**Recovery** **%**	**RSD%**	**Recovery** **%**	**RSD%**	**Recovery** **%**	**RSD%**	**Recovery** **%**	**RSD%**
BADGE·2H_2_O	87.8	1%	88.5	2%	88.2	1%	88.2	1.3%	88.0	3%	88.5	2%	88.3	2.5%	88.2	1.8%
BPF	78.2	3%	77.5	4%	77.7	3%	77.8	3.3%	77.7	1%	77.5	1%	77.6	1.0%	77.7	2.4%
BPE	77.8	2%	78.0	3%	77.8	3%	77.9	2.7%	78.2	3%	78.0	2%	78.1	2.5%	78.0	2.6%
BADGE·H_2_O	73.3	5%	72.8	3%	72.8	1%	73.0	3.0%	72.7	2%	73.5	5%	73.1	3.5%	73.0	3.2%
BPB	75.2	3%	74.8	6%	74.7	3%	74.9	4.0%	75.2	4%	74.8	3%	75.0	3.5%	74.9	3.8%
BADGE·2HCl	64.7	9%	64.5	14%	65.2	8%	64.8	10.3%	65.3	9%	65.8	4%	65.6	6.5%	65.1	8.8%
BADGE	56.7	9%	57.3	7%	56.8	7%	56.9	7.7%	56.8	8%	56.8	10%	56.8	9.0%	56.9	8.2%
**Recoveries Obtained for Fortification at 250 ng/mL Sample after the Procedure Shown in Figure 1.**																
**Bisphenol**	**Intra-Day Repeatability ^a^**						**Inter-Day** **Repeatability ^b^** **(*n* = 18)**		**Intra-Laboratory** **Reproducibility ^c^**						**Overall ^d^** **(*n* = 30)**	
**Name**	**Day 1 (*n* = 6)**		**Day 2 (*n* = 6)**		**Day 3 (*n* = 6)**				**Analyst 1 (*n* = 6)**		**Analyst 2 (*n* = 6)**		**Mean (*n* = 12)**			
	**Recovery %**	**RSD%**	**Recovery %**	**RSD%**	**Recovery (%)**	**RSD%**	**Recovery** **%**	**RSD%**	**Recovery** **%**	**RSD%**	**Recovery** **%**	**RSD%**	**Recovery** **%**	**RSD%**	**Recovery** **%**	**RSD%**
BADGE·2H_2_O	86.8	3%	87.7	1%	86.8	3%	87.1	2.3%	87.5	1%	88.3	2%	87.9	1.5%	87.4	2.0%
BPF	73.0	4%	73.2	4%	72.7	7%	73.0	5.0%	73.7	2%	72.8	3%	73.3	2.5%	73.1	4.0%
BPE	76.3	5%	75.8	4%	75.8	6%	76.0	5.0%	73.7	3%	75.7	4%	74.7	3.5%	75.5	4.4%
BADGE·H_2_O	71.2	7%	71.3	6%	70.7	9%	71.1	7.3%	71.3	2%	71.7	4%	71.5	3.0%	71.2	5.6%
BPB	72.2	10%	72.8	4%	73.7	5%	72.9	6.3%	72.8	4%	73.0	5%	72.9	4.5%	72.9	5.6%
BADGE·2HCl	67.3	9%	67.2	11%	66.7	8%	67.1	9.3%	66.8	11%	66.7	8%	66.8	9.5%	66.9	9.4%
BADGE	58.2	7%	57.8	4%	58.2	6%	58.1	5.7%	57.8	10%	57.3	14%	57.6	12.0%	57.9	8.2%

**^a^** Mean recovery % and RSD% for within-day results of batch of six samples per day (*n* = 6). **^b^** Mean recovery % and RSD% from 18 samples analyzed on three different days (*n* = 6 for each day). **^c^** Mean recovery % and RSD% from experiments conducted by two different analysts (*n* = 6 for each operator) and average results (*n* = 12). **^d^** average recovery % and RSD% from all experiments (*n* = 30).

## Data Availability

Data are contained within the article.

## Sample Availability

Samples of the compounds are not available.
